# Geminin Is Required for Zygotic Gene Expression at the Xenopus Mid-Blastula Transition

**DOI:** 10.1371/journal.pone.0038009

**Published:** 2012-05-25

**Authors:** Sarah L. Kerns, Kathryn M. Schultz, Kelly A. Barry, Tina M. Thorne, Thomas J. McGarry

**Affiliations:** 1 Feinberg Cardiovascular Research Institute, Northwestern University Feinberg School of Medicine, Chicago, Illinois, United States of America; 2 Department of Cell and Molecular Biology, Northwestern University Feinberg School of Medicine, Chicago, Illinois, United States of America; 3 Robert H. Lurie Cancer Center, Northwestern University Feinberg School of Medicine, Chicago, Illinois, United States of America; University of Colorado, Boulder, United States of America

## Abstract

In many organisms early development is under control of the maternal genome and zygotic gene expression is delayed until the mid-blastula transition (MBT). As zygotic transcription initiates, cell cycle checkpoints become activated and the tempo of cell division slows. The mechanisms that activate zygotic transcription at the MBT are incompletely understood, but they are of interest because they may resemble mechanisms that cause stem cells to stop dividing and terminally differentiate. The unstable regulatory protein Geminin is thought to coordinate cell division with cell differentiation. Geminin is a bi-functional protein. It prevents a second round of DNA replication during S and G2 phase by binding and inhibiting the essential replication factor Cdt1. Geminin also binds and inhibits a number of transcription factors and chromatin remodeling proteins and is thought to keep dividing cells in an undifferentiated state. We previously found that the cells of Geminin-deficient Xenopus embryos arrest in G2 phase just after the MBT then disintegrate at the onset of gastrulation. Here we report that they also fail to express most zygotic genes. The gene expression defect is cell-autonomous and is reproduced by over-expressing Cdt1 or by incubating the embryos in hydroxyurea. Geminin deficient and hydroxyurea-treated blastomeres accumulate DNA damage in the form of double stranded breaks. Bypassing the Chk1 pathway overcomes the cell cycle arrest caused by Geminin depletion but does not restore zygotic gene expression. In fact, bypassing the Chk1 pathway by itself induces double stranded breaks and abolishes zygotic transcription. We did not find evidence that Geminin has a replication-independent effect on transcription. We conclude that Geminin is required to maintain genome integrity during the rapid cleavage divisions, and that DNA damage disrupts zygotic gene transcription at the MBT, probably through activation of DNA damage checkpoint pathways.

## Introduction

In many metazoans embryonic development begins with a series of extremely rapid cleavage divisions that quickly produce a blastula containing thousands of cells. During this period development is under the control of maternal RNAs stored in the egg. Zygotic transcription is deferred until the mid-blastula stage, at a point called the mid-blastula transition (MBT) in Xenopus or the maternal-zygotic transition (MZT) in Drosophila [1]. Concomitant with activation of the zygotic genome, the cell cycle slows as gap phases are introduced between S and M phases. This pattern of development is thought to be an adaptation that rapidly provides enough cells to form a feeding larva, an important consideration for organisms with eggs that develop outside the mother's body. Rapid cell cycles have also been observed in mammalian embryos before gastrulation, suggesting that this mechanism of generating a large number of undifferentiated cells may be more widespread [2]. The mechanisms that switch on zygotic transcription at the MBT are incompletely understood. They may resemble those that induce the progeny of stem cells to withdraw from the cell cycle and execute a program of terminal differentiation.

The MBT occurs after a fixed number of cleavage divisions, after the 12^th^ cleavage in Xenopus and after the 14^th^ in Drosophila [3], [4]. In both organisms, the time of the MBT can be advanced or delayed by artificially increasing or decreasing ratio of nuclear DNA to cytoplasm [5], [6], [7]. These observations led to the model that the expanding mass of nuclear DNA titrates a cytoplasmic transcriptional repressor during the cleavage divisions, and that zygotic gene expression initiates when this factor is depleted. This putative cytoplasmic repressor has never been identified, but candidates include DNA N-methyl transferase 1 (Dnmt1) and the Drosophila protein *Tramtrack* [1].

A second model proposes that zygotic gene expression is controlled by a cell cycle-independent maternal “clock” that is activated at the time of fertilization. Recent microarray studies in Drosophila indicate that the transcription of most zygotic genes is not influenced by the nuclear/cytoplasmic ratio and depends only upon the time elapsed since fertilization [8]. The clock mechanism also controls the destruction of maternal RNAs, which occurs in parallel with zygotic gene activation. The RNA binding protein *Smaug* may be one component of this clock. In Drosophila, *Smaug* is required both for the initiation of zygotic transcription and for the changes in the cell cycle that occur at the MBT [9], [10]. *Smaug* triggers the destruction of specific mRNAs by recruiting them to the CCR4/POP2/NOT deadenylase complex [11]. *Smaug* may affect the timing of the cell cycle by targeting RNAs encoding Cdc25 and the mitotic cyclins. Cdc25 is the phosphatase that triggers mitotic entry by removing two inhibitory phosphate groups from the mitotic kinase Cdc2. In Drosophila, destruction of Cdc25 at the MBT delays mitotic entry and causes the cell cycle slowing [12].

A third model proposes that rapid cell cycling by itself inhibits zygotic transcription. According to this model, ongoing DNA replication or entry into mitosis aborts nascent zygotic transcripts. In both Xenopus and Drosophila, zygotic gene activation occurs precociously when cell cycling is inhibited with cycloheximide [13], [14]. Mitotic entry has also been directly shown to abort the transcription of a long messenger RNA [15].

Cell cycle checkpoint mechanisms can also affect zygotic transcription. Drosophila embryos that carry a mutation in Chk1, the effector kinase of the DNA replication checkpoint pathway, fail to slow the cell cycle and fail to express zygotic genes at the MBT [16], [17]. The Chk1 pathway delays entry into mitosis until DNA replication is complete. Initially these results were interpreted to mean that checkpoint-induced slowing of the cell cycle is necessary for transcription. It was found, however, that Chk1(−/−) embryos accumulate DNA damage in the form of double stranded breaks [18], and that zygotic gene expression is restored if the effector kinase of the DNA damage checkpoint pathway, Chk2, is also mutated [19]. This indicates that a Chk2-dependent DNA damage pathway can suppress zygotic transcription.

In this study we investigated how the unstable regulatory protein Geminin regulates the expression of zygotic genes at the mid-blastula transition. Geminin is thought to maintain cells in an undifferentiated state while they proliferate [20], [21]. Geminin is a bi-functional protein. It limits the extent of DNA replication to one round per cell cycle by binding and inhibiting the essential replication factor Cdt1 [22], [23], [24]. Geminin also inhibits transcription in several different tissues by binding and inhibiting various transcription factors and chromatin remodeling proteins [25], [26], [27]. Because Geminin regulates both the cell cycle and tissue-specific transcription, it has been suggested that the protein somehow coordinates cell division with cell differentiation.

We previously showed that Xenopus embryos lacking Geminin arrest in G2 phase just after the 13^th^ cell division, one cell cycle after the MBT is reached [28]. The cell cycle arrest is caused by activation of the Chk1-dependent DNA replication checkpoint. Geminin-depleted embryos retain viability for several hours but then disintegrate during gastrulation. Here we report that when Geminin is depleted the expression of most zygotic genes at the MBT is sharply reduced, and that the expression defect is a consequence of abnormal DNA replication. We conclude that Geminin is required to maintain genomic integrity during the early cleavage divisions, and that DNA damage disrupts zygotic transcription at the MBT, probably through activation of DNA damage-induced checkpoint mechanisms. We could not demonstrate that Geminin has a replication-independent effect on transcription.

## Results

### Geminin is required for early embryonic gene expression in Xenopus

**Figure 1:**
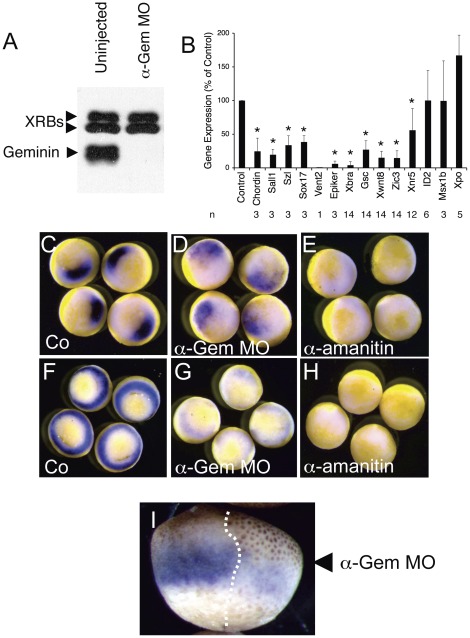
Geminin is Required for Zygotic Gene Expression. (A) Immunoblot showing complete depletion of Geminin by anti-Geminin morpholino oligonucleotides (α-Gem MOs). XRBs, cross-reacting bands serving as a loading control. Geminin runs as a tightly spaced doublet at ∼35 kD. (B) Real-time PCR (RT-PCR) data showing decreased expression of most genes in Geminin-depleted embryos at stage 10.5. The expression level if each RNA was normalized to the expression in control embryos (Co). n, number of independent experiments; asterisk indicates P<0.001 (paired t-test); NS, not significant. (C–H) In situ hybridization for Gsc (C–E) and Xbra (F–H) at stage 10.5 showing that depleting Geminin preserves the pattern of expression for both genes. Both cells of two-cell Xenopus embryos were left uninjected (Co) or injected with either anti-Gem MOs or α-amanitin. (I) Xbra expression is affected only in Geminin-depleted cells. One cell of a two-cell embryo was injected with anti-Geminin MOs (arrowhead) and in situ hybridization for Xbra was performed when the embryo reached stage 10.5. Note the markedly larger cells on the injected side (dotted line), indicating that the cells have stopped dividing.

To inhibit Geminin expression we injected two-cell Xenopus embryos with 32 ng of anti-Geminin morpholino oligonucleotides (α-Gem MOs), which cause a >95% reduction in the amount of Geminin protein through at least the gastrula stage (Fig. 1A, [28]). When the embryos reached the onset of gastrulation (Nieuwkoop stage 10.5) we compared the levels of a broad array of early transcripts in Geminin-depleted and control embryos by quantitative real-time PCR (RT-PCR). Geminin-depleted embryos showed a marked reduction in the expression of most early transcripts (Fig. 1B), including patterning genes of the Spemann organizer (Goosecoid (Gsc)), genes in the Wnt signaling pathway (Xwnt8, Sizzled (Szl)), genes in the BMP signaling pathway (Chordin (Chrd), Vent2), and genes expressed in the three germ layers (Brachury (Xbra, mesoderm), epidermal keratin (Epiker, epidermis), Zic3 (neural plate), and Sox17 (endoderm). The transcript levels of two genes expressed before the MBT, Nodal-related 5 (Xnr5) [29] and ID2 [30], were not significantly affected. Only two genes, Msx1b and Xenopus-posterior (Xpo), showed preserved induction in Geminin-depleted embryos. These results show that Geminin depletion causes widespread defects in zygotic transcription but has less of an effect on genes expressed before the MBT. The decrease in gene expression is not specific to any particular signaling pathway, organ system, or germ layer.

We next used in situ hybridization to examine how Geminin depletion affects the pattern of expression of Gsc and Xbra, two genes with a distinctive and well-known embryonic localization. Gsc is normally expressed in the Spemann organizer on the dorsal side of the embryo close to the blastopore lip (Fig. 1C, [31]). When Geminin is depleted, Gsc expression is still detected on the dorsal side near the blastopore lip but expression is not as tightly localized (Fig. 1D). Xbra is normally expressed in a circumferential band of cells that give rise to the embryonic mesoderm (Fig. 1F, [32]). When Geminin is depleted, Xbra expression is barely detectable but the small amount of residual expression shows the normal circumferential staining pattern (Fig. 1G). Neither Gsc nor Xbra expression could be detected in embryos injected with the RNA polymerase II inhibitor α-amanitin (Figs. 1E and 1H). These results indicate that Geminin depletion affects the level of gene expression but not the pattern or the timing.

When only one cell of the two-cell embryo was injected with antisense oligo, Xbra expression was lost only on the injected side (Fig. 1I). This indicates that the Geminin-depleted embryos survive until the stage in development when zygotic gene expression begins, and that the effects of Geminin depletion are cell-autonomous (see below). TUNEL staining indicated that the Geminin-depleted cells had not undergone apoptosis (Fig. S1, A–C). The Geminin-depleted cells are noticeably larger than uninjected cells at this stage of development, indicating that they have undergone a cell cycle arrest (Fig. 1I, compare cell size on either side of the dotted line).

**Figure 2:**
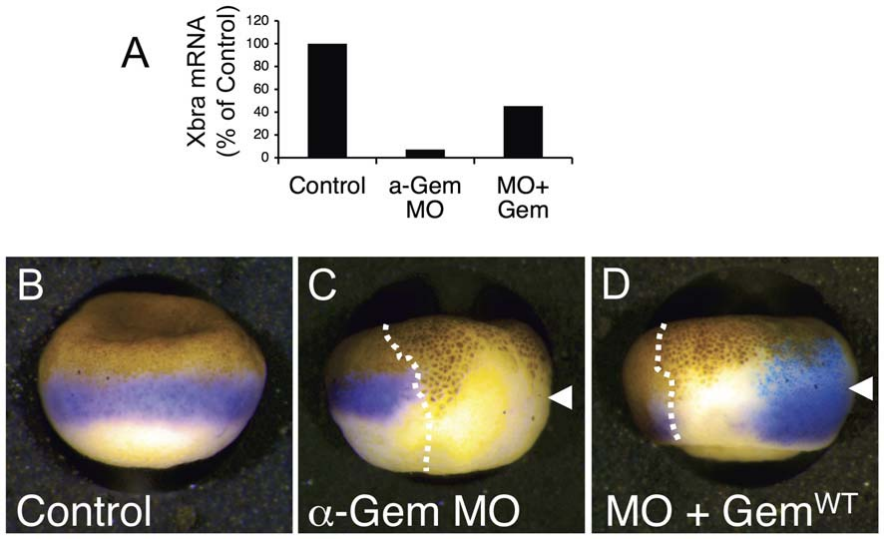
Geminin Expression Restores Zygotic Gene Expression. (A) Two-cell embryos were left uninjected (Control), injected on both sides with anti-Geminin MOs, or injected with anti-Geminin MOs followed by RNA encoding a MO-resistant version of Geminin^WT^. At stage 10.5 the amount of Xbra RNA was determined by RT-PCR. The average of two measurements is shown. (B–D) Same as (A), except only one cell of a 2-cell embryo was injected (arrowheads) and LacZ RNA was co-injected with Geminin^WT^ RNA to serve as a lineage tracer. At stage 10.5 Xbra RNA was visualized by in situ hybridization (purple) and ß-galactosidase activity was visualized by staining with Xgal (blue). The dotted lines indicate the border between the uninjected half and the injected half.

Xbra expression is restored by injecting Geminin-depleted embryos with synthetic Geminin RNA that has been mutated so that it does not interact with the anti-Geminin MOs (Fig. 2A, [28]). This indicates that the expression defect results specifically from the loss of Geminin and not from an off-target effect of the oligonucleotides. Xbra expression was not completely restored to the normal level, probably because Geminin RNA does not diffuse as freely throughout the embryo as the antisense oligonucleotides. To confirm this, we repeated the experiment and co-injected β-galactosidase RNA as a lineage tracer to demarcate the injected area. We found that Xbra expression was fully restored near the injection site where Geminin was expressed (Figs. 2B–D).

### Geminin's Effects are Cell Autonomous

Gsc and Xbra are induced by extracellular signals that emanate from the Nieuwkoop signaling center on the dorsal side of the embryo [33]. The loss of Gsc and Xbra expression in Geminin-deficient embryos could reflect either a failure of the Nieuwkoop center to generate these signals or a failure of uncommitted cells to respond to them. Our observation that residual Gsc and Xbra are still expressed in a spatially and developmentally specific pattern suggests that the signals inducing Gsc and Xbra expression are intact. Furthermore, when only one cell of a two-cell embryo was injected, we occasionally observed small islands of Xbra-expressing cells near the border zone that were surrounded by Xbra non-expressing cells (not shown). This pattern is more consistent with a cell-autonomous defect in responding to extracellular induction signals.

**Figure 3:**
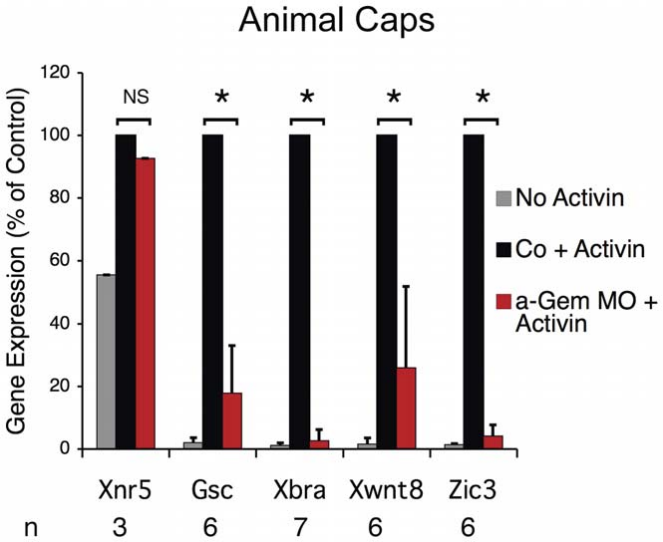
Geminin's Effects on Gene Expression are Cell-Autonomous. Animal caps were dissected from control (black bars) or Geminin-depleted (red bars) embryos at the early blastula stage (Nieuwkoop stage 8) and incubated in 20 ng/ml Human Activin A. When companion embryos reached stage 10.5, RNA was isolated from the caps and message levels were measured by RT PCR as described above. Gray bars, message level in control caps not treated with activin; n, number of measurements; asterisk indicates P<0.002 (paired t-test); NS, not significant.

To confirm this, we dissected undifferentiated animal caps from Geminin-depleted or control embryos and exposed them to recombinant human Activin A, a mesoderm-inducing soluble ligand protein that reproduces the signals coming from the Nieuwkoop center. In control animal caps, activin treatment induces Gsc expression about 40-fold and Xbra expression about 100-fold (Fig. 3, compare gray and black bars). In Geminin-depleted caps, activin-induced Gsc expression is reduced 5-fold and Xbra expression is reduced 50-fold (Fig. 3, compare black and red bars), mirroring the results seen in intact embryos. Similar results were obtained with Xwnt8 and Zic3 expression. Furthermore, Xnr5 expression was not affected by Geminin depletion, as was observed in intact embryos. These results indicate that Geminin's effects on zygotic gene expression are cell-autonomous.

### Gene Expression Defects are due to Over-activity of Cdt1

**Figure 4:**
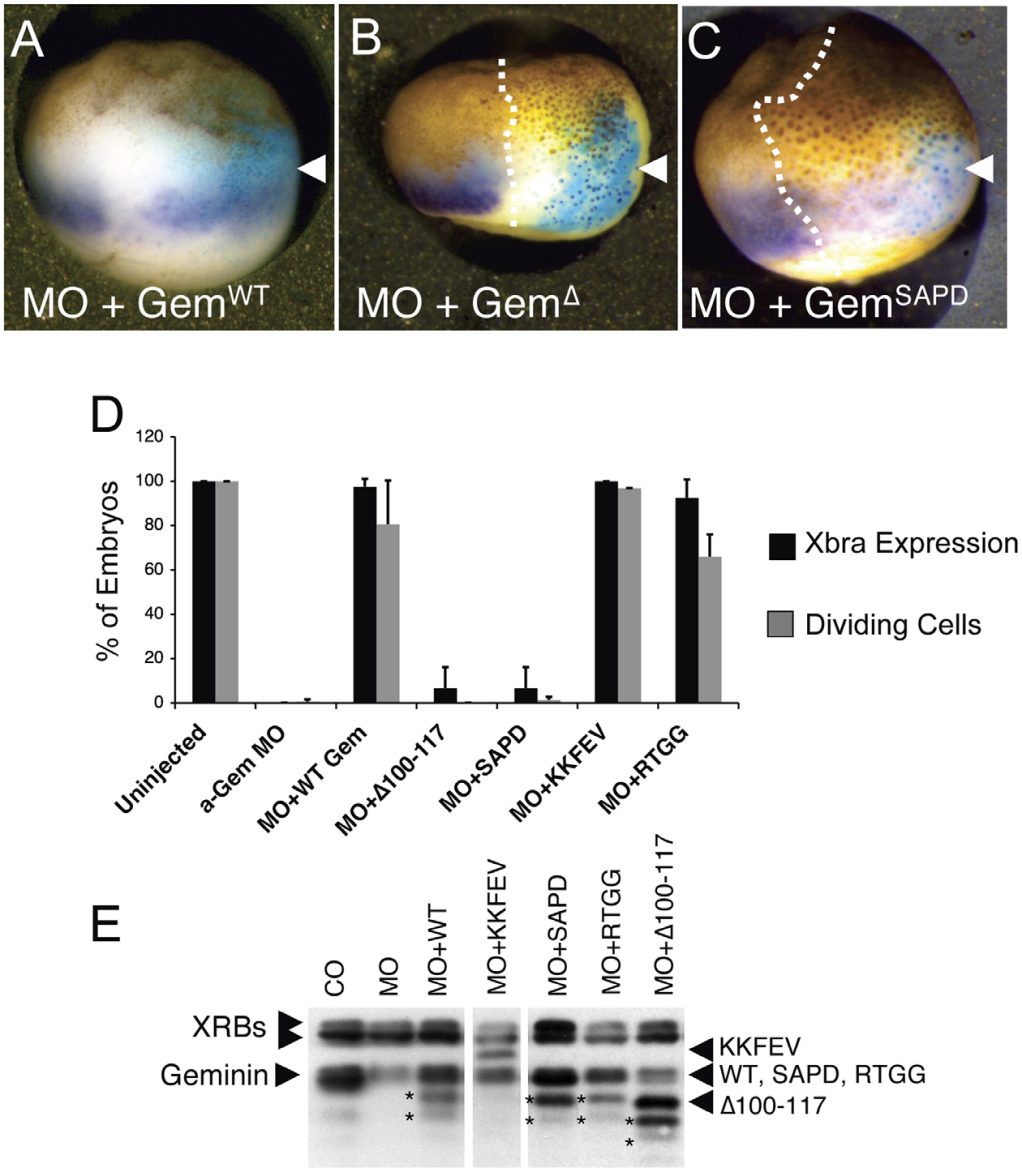
Geminin Mutants that don't bind Cdt1 fail to restore Xbra Expression. (A–C) One cell of a two-cell embryo was injected with anti-Gem MOs and RNA encoding a MO-resistant version of either Geminin^WT^ or various Geminin mutants along with LacZ RNA as a lineage tracer. At stage 10.5 Xbra RNA was visualized by in situ hybridization (purple) and ß-galactosidase activity was visualized by staining with Xgal (blue). The white dotted line indicates the border between the injected and the uninjected side. (D) Graph showing percentage of embryos showing restoration of Xbra expression (black bars) and the percentage of embryos showing suppression of the cell cycle arrest (gray bars) in the injected area. The average and standard deviation of three measurements is shown. (E) Immunoblot showing equivalent expression of all Geminin mutants. Note that Geminin was not completely depleted (lane 2) because only one side of the embryo was injected with MOs. XRBs, cross-reacting bands serving as a loading control; arrowheads, Geminin mutants; asterisks, Geminin degradation products.

It has been reported that Geminin represses gene expression through interactions with homeobox (Hox) proteins, sine-oculis (Six) transcription factors, the *Polycomb* protein Scmh1, the BMP signaling pathway, and the chromatin remodeling protein Brg1 [25], [26], [27], [34], [35]. We next tested whether reduced zygotic gene expression in Geminin depleted embryos was linked to an effect on DNA replication or the result of an independent action of Geminin on transcription. Geminin regulates DNA replication by binding and inhibiting the essential replication factor Cdt1. We previously generated two Geminin mutants (Geminin^SAPD^ and Geminin^Δ100–117^) that do not bind Cdt1 and do not inhibit DNA replication when added to extracts of Xenopus eggs [36]. To see if either mutant could rescue Xbra expression, we injected two-cell embryos with α-Gem MOs and 30 pg of RNA encoding either WT Geminin or one of the Cdt1 binding mutants, along with ß-galactosidase RNA as a lineage tracer. At stage 10.5, neither non-binding mutant restored Xbra expression in any of the embryos, while Geminin^WT^ restored expression in almost all (Fig. 4A–C, D). We also tested two other Geminin missense mutants (Geminin^KKFEV^ and Geminin^RTGG^) that map to the same region as Geminin^SAPD^ and Geminin^Δ100–117^ yet bind Cdt1 normally [36]. These mutants were also able to rescue Xba expression. The ability of each mutant to restore Xbra expression was directly correlated with its ability to bind Cdt1 and to reverse the cell cycle arrest caused by depleting Geminin (Fig. 4D, see below). Immunoblotting confirmed that each mutant was expressed at the same level (Fig. 4E). These results indicate that Geminin must be able to bind Cdt1 in order to rescue zygotic gene expression.

**Figure 5:**
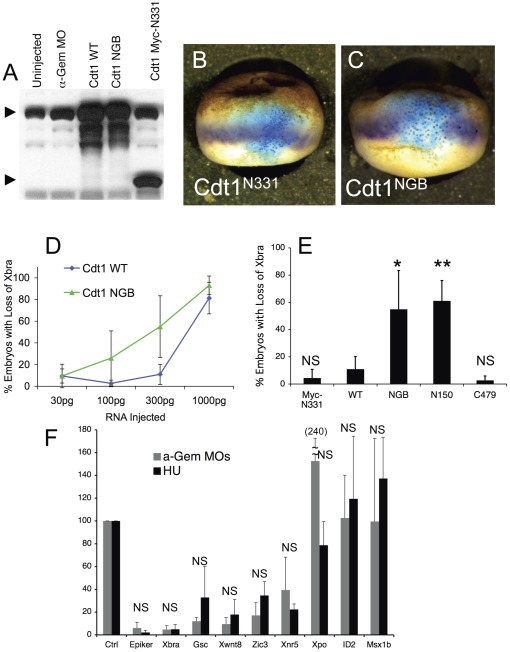
DNA Damage Reproduces the Effects of Depleting Geminin. Two-cell Xenopus embryos were injected on one side with RNA encoding Cdt1^WT^ or different Cdt1 mutants along with LacZ RNA as a lineage tracer then analyzed at stage 10.5. (A) Immunoblot showing equivalent expression of Cdt1 WT, Cdt1NGB, and myc-Cdt1N331. (B, C) Typical appearance of embryos injected with myc-Cdt1^N331^ or Cdt1^NGB^. (D) Percentage of embryos with reduced Xbra expression in the injected area as a function of the amount of Cdt1^WT^ or Cdt1^NGB^ RNA injected. (E) Percentage of embryos showing reduced Xbra expression after injection of 300 pg of RNA encoding different Cdt1 mutants. The average and standard deviation of three measurements is shown. An asterisk indicates P<0.01; a double asterisk indicates P<0.001 compared to injection of Cdt1^WT^. (F) Two-cell embryos were injected on both sides with anti-Geminin MOs (gray) or incubated in 20 mM hydroxyurea (black). At stage 10.5 RNA levels were measured by RT-PCR. The average value of three independent experiments is graphed. NS, not significant.

We next tested whether over-expressing Cdt1 by itself reproduces the defects in transcription seen with Geminin depletion. We previously described a non-Geminin binding mutant of Cdt1 (Cdt1^NGB^) that retains its full activity as a replication factor, and a different non-binding mutant that is catalytically inactive (Myc-Cdt1^N331^) [37]. We injected one cell of two-cell Xenopus embryos with different concentrations of RNA encoding Cdt1^WT^, Cdt1^NGB^, or Myc-Cdt1^N331^ along with ß-galactosidase RNA as a lineage tracer. Immunoblotting confirmed that each Cdt1 mutant was expressed at the same level (Fig. 5A). Expressing either Cdt1^WT^ or Cdt1^NGB^ inhibited Xbra expression, while expressing the catalytically inactive Myc-Cdt1^N331^ had no effect (Fig. 5B–C). The non-Geminin binding mutant was more potent at suppressing Xbra expression than the wild-type protein (Fig. 5D), as was a stabilized form of Cdt1 (Cdt1^N150^) that does not get proteolyzed during S phase (Fig. 5E, [37]). Over-expressing Cdt1^C479^, a C-terminal deletion mutant that binds Geminin but is inactive as a replication factor [37] does not affect Xbra expression (Fig. 5E). TUNEL assays showed that cells expressing the most potent mutant, Cdt1NGB, had not undergone apoptosis (Fig. S1D). We conclude from these experiments that the reduced gene expression in Geminin depleted embryos is a consequence of over-activity of Cdt1. Cdt1 is required for the assembly of pre-replication complexes during S phase but has no other known function [38]. The results of Cdt1 over-expression indicate that the effect of Geminin on Xbra transcription is caused by over-replication of the DNA.

### Hydroxyurea Reproduces the Transcription Defects caused by Geminin Deletion

We next tested whether chemical DNA damaging agents have a similar effect on transcription as Geminin depletion. Hydroxyurea (HU) blocks DNA replication by inhibiting the enzyme ribonucleotide reductase and depleting the pool of deoxyribonucleotide triphosphates. Treating Xenopus embryos with HU activates both the DNA replication checkpoint and DNA damage checkpoint pathways, and the cells stop dividing immediately after the 12^th^ cell division [28], [39]. HU-treated Xenopus embryos show the same pattern of transcriptional abnormalities as Geminin-depleted embryos: the expression of most zygotic genes is reduced while the expression of Xpo, ID2, and Msx1b is preserved (Fig. 5F). TUNEL staining showed that some of the cells in HU-treated embryos had undergone apoptosis (Fig. S1E), but apoptosis did not seem extensive enough to fully account for the changes in gene expression (compare Fig. S1 A and E). This indicates that a DNA damaging agent has the same effect on transcription as Geminin depletion.

### Suppressing the Cell Cycle Arrest does not Restore Xbra Expression

**Figure 6:**
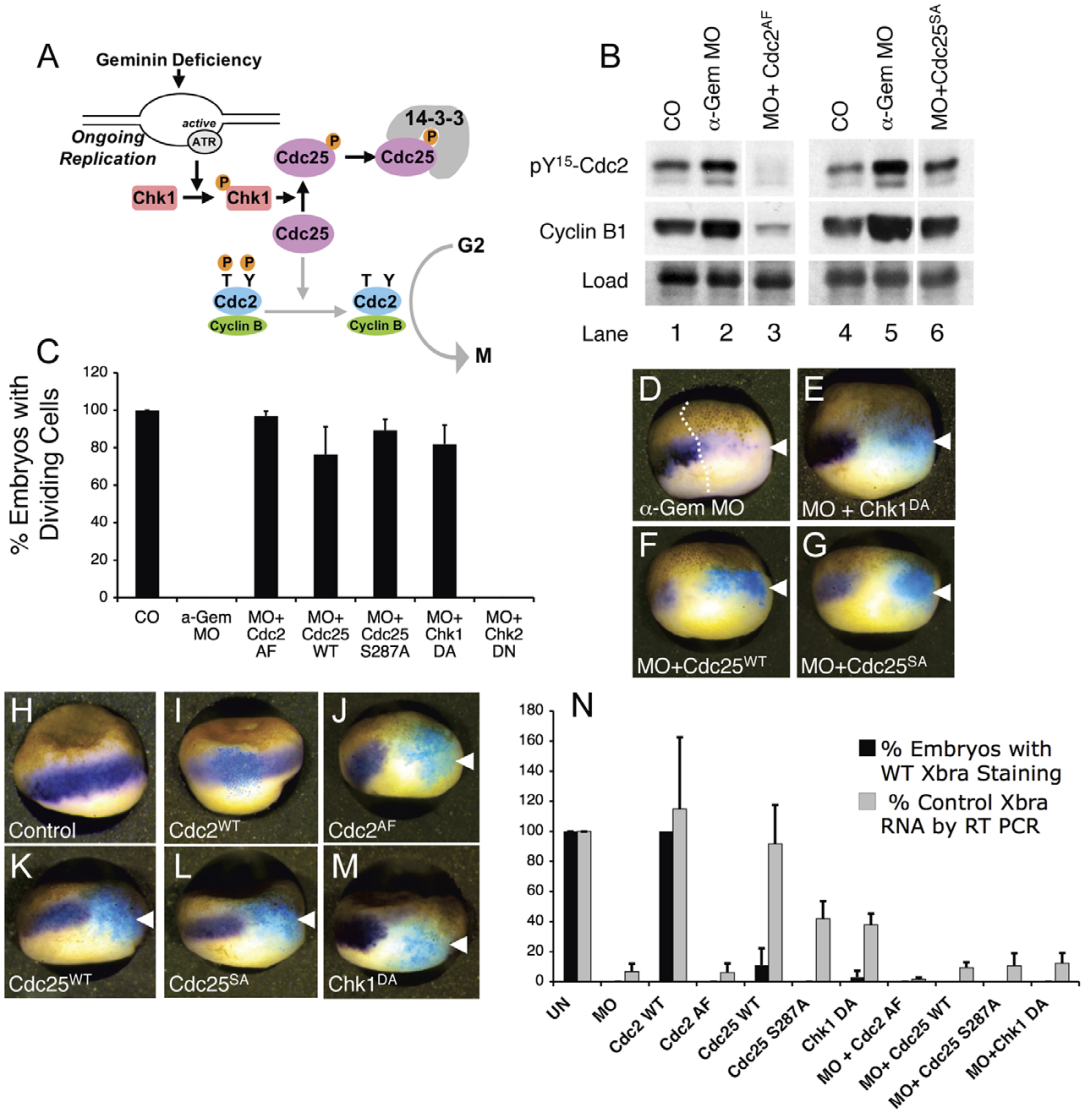
Suppressing the Cell Cycle Arrest does not Restore Xbra Expression. (A) The signaling pathway that controls entry into mitosis. See text for details. (B) Arrested Geminin-deficient cells exhibit increased phosphorylation of Cdc2 on Y^15^ and increased levels of B-type cyclins; and these changes are reversed by over-expressing either Cdc2^AF^or Cdc25^S287A^. Two-cell embryos were left uninjected (CO), injected on both sides with anti-Gem MOs (a-Gem MOs), or injected with anti-Gem MOs followed by RNA encoding Cdc2^AF^ or Cdc25^S287A^. At stage 10.5, phosphorylated Cdc2 and cyclin B1 levels were determined by immunoblotting. Load, cross-reacting band serving as a loading control. (C) The cell cycle arrest is reversed by over-expressing Cdc25^WT^, Cdc25^S287A^, Cdc2^AF^, or Chk1^DA^. One cell of a two-cell embryo was injected with anti-Geminin MOs and RNA encoding the indicated proteins. The plot shows the percentage of embryos in which cell division was restored in the injected area at stage 10.5. (D–M) One cell of a two-cell embryo was injected with anti-Gem MOs and/or RNA encoding the indicated proteins. LacZ RNA was co-injected as a lineage tracer. At stage 10.5 Xbra RNA was visualized by in situ hybridization (purple) and beta galactosidase activity was visualized by staining with Xgal (blue). (N) Both sides of a two-cell embryo were injected with anti-Geminin MOs and/or RNA encoding the indicated proteins. At stage 10.5, RNA was extracted and the amount of Xbra mRNA was measured by RT-PCR (gray bars). In a parallel experiment, one cell of a 2-cell embryo was injected in the same way along with LacZ as a lineage tracer. At stage 10.5 Xbra was visualized by in situ hybridization and the percentage of embryos showing normal Xbra expression was determined (black bars).

**Figure 7:**
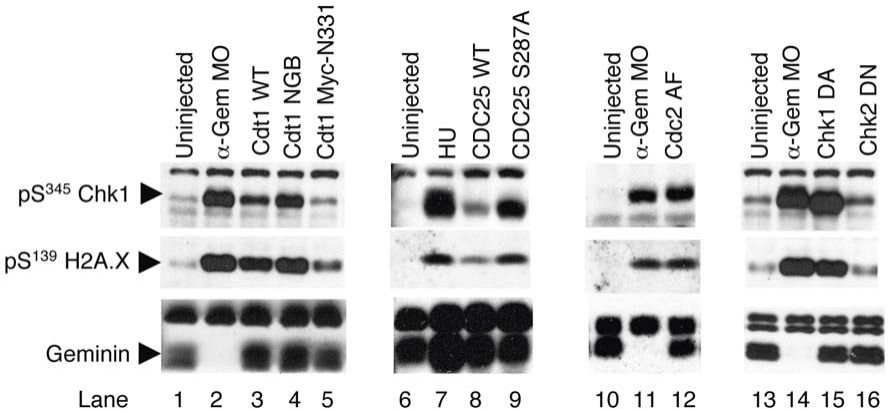
Treatments that Suppress Zygotic Gene Expression Cause DNA Damage. Two-cell Xenopus embryos were injected on both sides with anti-Geminin MOs and/or RNA encoding the indicated proteins. The levels of S^345^-phosphorylated Chk1, S^139^-phosphoryated H2A.X, and Geminin were determined by immunoblotting.

We previously showed that Geminin depletion arrests blastula cells in the G2 phase of the cell cycle because over-replication of the DNA activates the Chk1-dependent DNA replication checkpoint pathway (Fig. 6A, [28], [37], [40]). Activation of the checkpoint is manifest by the appearance of Chk1 phosphorylated on serine-345 (see Fig. 7, lanes 1–2). Chk1 arrests cells in G2 phase by phosphorylating Cdc25 on serine-287, causing it to bind 14-3-3 proteins and be sequestered in the cytoplasm where it cannot activate the mitotic kinase Cdc2 (Fig. 6A, [41], [42]). Because of the G2 arrest, Geminin-depleted embryos have visibly larger cells than control embryos at the late blastula stage (see Figs. 1I, 2C, and 6D). They also exhibit two molecular signatures of G2 phase: excessive accumulation of mitotic cyclins and complete phosphorylation of Cdc2 on tyrosine-15 (Fig. 6B, compare lanes 1–2 and 4–5; [28]). We previously showed that the G2 arrest could be bypassed by over-expressing constitutively active forms of Cdc25 or Cdc2 (Cdc25^S287A^ or Cdc2^AF^, respectively) or by over-expressing a dominant negative mutant of Chk1 (Chk1^DA^) [28].

To test whether the gene expression defects in Geminin-depleted embryos are caused by the cell cycle arrest, we suppressed the checkpoint pathway by injecting 2-cell embryos with both α-Gem MOs and RNA encoding either Cdc2^AF^, Cdc25^WT^, Cdc25^S287A^, or Chk1^DA^. Over-expression of each protein efficiently reversed the cell cycle arrest caused by Geminin depletion, as indicated by the disappearance of large arrested cells near the injection site (Fig. 6C) and a reduction in the amounts of cyclin B1 and phosphorylated Cdc2 (Fig. 6B, compare lanes 2–3 and 5–6; data not shown; and [28]). Although the Geminin-deficient cells continued to divide past the point they would normally arrest, there was virtually no restoration of Xbra expression as judged by either RT-PCR or by in situ hybridization (Figs. 6D–6G, 6N). We conclude that reduced expression of zygotic genes in Geminin-depleted embryos is not a direct consequence of a G2 cell cycle arrest. Moreover, we found that injecting normal embryos with RNA encoding either Cdc25^S287A^, Cdc2^AF^, or Chk1^DA^ by itself caused a drastic reduction in Xbra expression even when the Geminin concentration was normal (Figs. 6H–M). The reduction was evident both by RT-PCR and by in situ hybridization (Fig. 6N). The in situ hybridization data show a more striking decrease in Xbra expression because the injected RNA does not diffuse freely throughout the embryo, as indicated by the limited area of X-gal staining. Injection of Cdc2^WT^ RNA, which does not suppress the checkpoint [28], had no effect (Figs. 6I and 6N). Expression of Cdc2^AF^ also caused a significant decrease in the expression of Gsc, Xwnt8, and Zic3 as measured by RT PCR (Figure S2). TUNEL staining showed that expressing Cdc2^AF^ or Cdc25^SA^ did not cause widespread cellular apoptosis (Fig. S1 F–G). Expressing Chk1^DA^ did cause some apoptosis of cells in the injected area (Fig. S1H). This “toxic” effect of expressing Chk1^DA^ may be nonspecific because much more Chk1^DA^ RNA was injected compared to Cdc2^AF^ or Cdc25^SA^ RNA (5000 pg vs. 150 pg). Considering all the results, we conclude that an intact DNA replication checkpoint pathway is also required for zygotic gene expression.

### Geminin Deletion Generates Double Stranded Breaks

Previous work in Drosophila has demonstrated that DNA-damaging agents or mutations in the Chk1 pathway generate double-stranded breaks, and that these forestall zygotic gene expression at the MZT through activation of the Chk2-dependent DNA damage checkpoint pathway [19]. We thought this mechanism might account for the gene expression defects in both Geminin-depleted embryos and in embryos where the Chk1 pathway has been suppressed. To check directly for the presence of DNA damage, we blotted embryo extracts with phosphospecific antibodies raised against S^139^-phosphorylated H2A.X, a modified histone that accumulates at double-stranded breaks [43], [44]. Geminin-depleted embryos exhibited robust phosphorylation of H2A.X on S^139^, indicating that Geminin deletion generates double stranded breaks (Fig. 7, lanes 2, 11, and 14). We also detected H2A.X phosphorylation in embryos injected with RNA encoding Cdt1^WT^ or Cdt1^NGB^ (lanes 3–4); embryos in which the DNA replication checkpoint was bypassed by injecting RNA encoding Cdc2^AF^, Cdc25^WT^, Cdc25^S287A^, or Chk1^DA^ (lanes 8, 9, 12, and 15); and embryos treated with hydroxyurea (lane 7). Thus, every treatment that inhibited zygotic gene expression also induced double stranded breaks.

**Figure 8:**
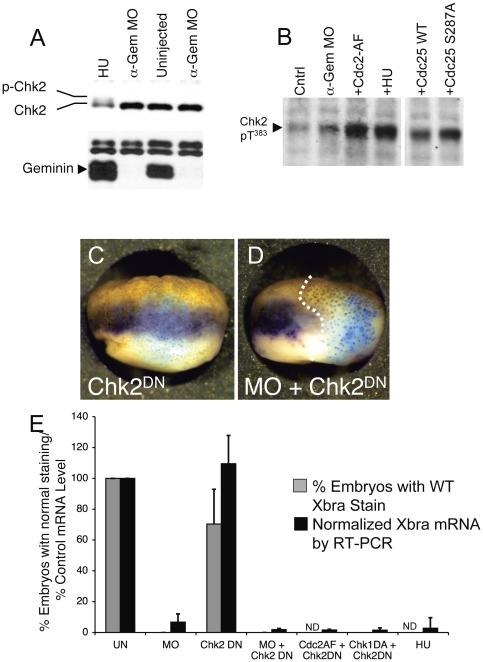
Suppressing the DNA Damage Checkpoint does not restore Zygotic Gene Expression. (A, B) Hydroxyurea treatment or suppression of the DNA replication checkpoint pathway causes activation of the DNA damage checkpoint kinase Chk2. Two-cell Xenopus embryos were left uninjected, treated with hydroxyurea, or injected on both sides with anti-Geminin MOs or RNA encoding the indicated proteins. At stage 10.5, immunoblots were performed for total Chk2, Geminin, and T^383^-phosphorylated Chk2. (C, D). One cell of a 2-cell embryo was injected with RNA encoding Chk2^DN^ with or without anti-Geminin MOs, along with ß–galactosidase RNA as a lineage trader. At stage 10.5, in situ hybridization was performed to visualize Xbra expression (purple) and X–gal staining was performed to visualize ß–galactosidase expression (blue). (E) 2-cell embryos were injected on both sides with anti-Geminin MOs and/or the indicated RNAs. At stage 10.5, Xbra RNA expression as measured by in situ hybridization (gray bars) and RT-PCR (black bars). ND, not determined.

Chk2 activation requires phosphorylation of the activation loop, which can be detected by either an electrophoretic mobility shift (Fig. 8A) or by immunoblotting with antibodies to phosphorylated T^383^ (Fig. 8B, [45], [46], [47]). To see if the Chk2-dependent pathway had been activated, we blotted embryo extracts with antibodies to either total Chk2 or T^383^-phosphorylated Chk2. We detected robust phosphorylation of Chk2 in hydroxyurea-treated embryos, Cdc2^AF^-injected embryos, and Cdc25^S287A^-injected embryos (Fig. 8A–B). In contrast, there was little if any Chk2 phosphorylation in Geminin-depleted embryos.

We also tested whether inhibition of the Chk2 pathway would restore Xbra expression to Geminin-depleted embryos by injecting embryos with RNA encoding a dominant negative form of Chk2 kinase, Chk2^DN^ [48]. Chk2^DN^ did not restore Xbra expression in Geminin-depleted embryos, whether measured by in situ hybridization (Fig. 8D) or by RT-PCR (Fig. 8E). Chk2^DN^ also failed to restore Xbra expression to embryos that had been injected with RNA encoding Cdc2^AF^, Cdc25^S287A^, or Chk1^DN^ (Fig. 8E). Immunoblots showed that Chk2^DN^ protein was expressed at about the same level as endogenous Chk2 (not shown), which may have been insufficient to completely inhibit the kinase. Injecting embryos with anti-Chk2 morpholino oligonucleotides did not affect the Chk2 protein level, and injecting embryos with anti-Chk2 antibodies caused nonspecific toxic effects (not shown). Incubating Geminin-depleted embryos in medium containing the Chk2 inhibitor 2-4-4-chlorophenoxyphenyl benzimidazole 5-carboxamide did not restore Xbra expression (not shown). In summary, by using a variety of methods, we could not demonstrate that inhibiting Chk2 activity restores gene expression in Geminin-depleted embryos. Embryos expressing Chk2^DN^ had normal Xbra expression (Fig. 8C and E), did not exhibit increased cellular apoptosis (Fig. S1I) and developed normally to the tadpole stage (not shown).

It has recently been reported that Geminin knockdown enhances the expression of some embryonic genes as much as 10-fold [35]. We tested the expression of four of the most highly induced genes: Epiker, Msx1b, ID2, and Xpo. With Geminin depletion, expression of these genes either did not change (Msx1b, ID2) or went down (Epiker) (Fig. 1B). Xpo RNA was induced about ∼2-fold but this result was extremely variable and not statistically significant. The other workers injected less anti-Geminin MO than we did (5 ng vs. 32 ng), so we tested whether this difference in technique was responsible for the conflicting results. At the lower dose of α-Gem MO, Geminin was only partially depleted (∼75%); embryos rarely exhibited a cell cycle arrest (24±14% of embryos); and that levels of S^345^-phosphorylated Chk1 and S^193^-phosphorylated H2A.X were lower compared to embryos where Geminin was completely depleted (Fig. S3, right panel). At the lower dose, the expression of most measured genes was intermediate between the level in untreated embryos and the level in fully depleted embryos (Fig. S3, left panel), This result argues against the possibility that there are threshold effects of depleting Geminin on gene expression. We suspect that the differences between our results and those previously published are mostly due to variability of gene expression in outbred frog populations and slight differences in the time when the embryos were collected.

## Discussion

In this paper we show that Geminin-deficient Xenopus embryos have a widespread defect in zygotic gene expression at the mid-blastula transition. The transcription of most genes is sharply reduced, while the expression of genes activated before the MBT, e.g. Xnr5 and ID2, is maintained. Zygotic induction was preserved for only two genes, Msx1b and Xpo. Geminin depletion apparently does not interfere with the timing mechanisms that cause zygotic transcription to initiate after the 12^th^ cell division, since a small residual level of Gsc and Xbra transcription occurs in the right place and at the right time. This is consistent with prevailing models, since Geminin loss does not affect the nuclear to cytoplasmic ratio, nor would it be expected to affect intrinsic maternal clock mechanisms that initiate at fertilization. Rather, Geminin loss affects the degree to which zygotic genes are expressed. Defects in zygotic gene expression may contribute to the early embryonic mortality of Geminin-depleted Xenopus and mouse embryos [28], [49], [50]. Geminin-deficient Drosophila embryos survive to larval stages, perhaps because of a maternal supply of Geminin RNA and protein [51].

The most parsimonious explanation of our results is that Geminin depletion inhibits zygotic gene expression because it leads to DNA damage. We previously showed that Geminin deletion causes a partial second round of DNA replication within a single S phase, and others have shown that Geminin knockdown in cultured cells generates both double stranded breaks and single stranded regions [52], [53], [54], [55]. Here we find that we can reproduce the effects of Geminin depletion on transcription by over-expressing Cdt1, a protein negatively regulated by Geminin and whose only known function is in DNA replication. This strongly suggests that over-replication of the DNA is responsible for the suppression of transcription. We can also suppress transcription by treating embryos with hydroxyurea or by bypassing the Chk1-dependent DNA replication checkpoint. DNA damage is a common feature of all the treatments that suppress transcription, as evidenced by the appearance of H2A.X phosphorylated on S^139^, a marker for double stranded breaks [43], [44]. It seems unlikely that these abnormal DNA structures by themselves abort nascent zygotic transcripts because in the absence of Geminin the total amount of re-replicated DNA is less than a few percent [28], [37].

It seems more likely that DNA damage inhibits transcription through activation of a checkpoint mechanism. It has previously been shown in Drosophila that activation of the Chk2-dependent DNA damage checkpoint pathway can block zygotic gene expression [19]. The Chk2 pathway can be activated either directly by injecting DNA damaging chemicals or indirectly by mutating the Chk1 protein, which causes double stranded breaks when cells enter mitosis before DNA synthesis is finished [18]. The Chk2 pathway also seems to inhibit zygotic transcription in Xenopus, since either incubating embryos in hydroxyurea or bypassing the Chk1 pathway also induces DNA damage, activates Chk2, and blocks zygotic gene activation. The definitive test of this model would be to show that inhibition of Chk2 signaling restores zygotic gene expression. In Drosophila this was accomplished by using Chk2(−/−) mutant strain, but unfortunately such a strain is not available in Xenopus. Because we could not reliably inhibit Chk2 in Xenopus embryos, the conclusion that Chk2 activation inhibits zygotic gene expression must remain somewhat tentative. The DNA damage response pathways may be more complex in vertebrates than they are in Drosophila, and it may not be possible to overcome them simply by inhibiting Chk2. Depleting Geminin does not consistently induce Chk2 phosphorylation, suggesting that a different checkpoint pathway could be involved. In vertebrate cells, a separate DNA-damage response pathway involving p38-MAPK arrests the cell cycle and promotes cellular survival [56]. Although MAPK signaling pathways are functional during early Xenopus development [57], we could not consistently demonstrate activation of p38-MAPK in Geminin-depleted embryos, nor could we restore Xbra expression by inhibiting this pathway (not shown). An alternate explanation is that ongoing DNA replication changes the structure of the chromatin template in some way that is not conducive to efficient transcription.

We could not demonstrate that Geminin has an independent effect on zygotic transcription that is separate from its effect on DNA replication. It has been previously reported that Geminin represses gene expression at the MBT through an interaction with the Polycomb complex PRC2 [35]. This conclusion was based on the finding that over-expressing the deletion mutant Geminin^Δcoil^ repressed the expression of several genes (including Xbra) and that the repression was relieved by injecting antisense MOs to the PRC2 subunits Suz12 or Ezh2. Geminin^Δcoil^ does not affect DNA replication [22], [34], so this mechanism appears to be separate from the one discussed here. We did not consistently observe over-expression or premature expression of any gene when Geminin was depleted (Fig. 1B and not shown). Furthermore, in our hands inhibiting PRC2 function by injecting an antisense MO to the Suz12 subunit (using the same conditions as in [35]) had no effect on development to the tadpole stage (Nieuwkoop stage 44), nor did it affect Xbra expression in control or Geminin-depleted embryos (not shown). Geminin has also been previously shown to inhibit transcription in several different tissues by interacting with various transcription factors and chromatin remodeling proteins [25], [26], [27], [34]. These include *Homeobox* (*Hox*) transcription factors; Brg1, the catalytic subunit of a SWI/SNF chromatin-remodeling ATPase; and the *Polycomb* group protein Scmh1, a sub-stoichiometric subunit of the PRC1 chromatin remodeling complex that maintains previously established transcriptional repression [26]. These regulatory interactions occur at later stages in development that Geminin-depleted embryos never reach. We found that over-expression of Scmh1 had no discernible effect on early Xenopus development or on Xbra expression in control or Geminin-depleted embryos (not shown). The significance of the Geminin-Scmh1 interaction remains a subject for further investigation.

The absolute requirement for Geminin during early development stands in stark contrast to its apparent dispensability at later stages. Deleting Geminin from mouse T lymphocytes, leukocytes, or neural stem cells has little effect on the production or function of these cells, even though it may cause subtle cell cycle defects [58], [59], [60], [61]. The exquisite sensitivity of early embryonic cells to Geminin depletion may reflect some peculiar aspect of their cell cycle. Depleting Geminin from hematopoietic stem cells has a striking effect on the types of cells that they give rise to: it abolishes the production of red blood cells but greatly enhances the production of megakaryocytes [60]. It is not yet known whether this is a consequence of a greater sensitivity of red cell precursors to replication damage or whether Geminin has a replication-independent effect on the differentiation of Megakaryocyte-Erythrocyte Progenitors.

## Materials and Methods

### Ethics Statement

All animal work was performed according to the protocol approved by the Northwestern University Animal Care and Use Committee (Protocol # 2009-0911).

### Xenopus

Adult male and female frogs were purchased from Nasco. Induction of ovulation and in vitro fertilization were carried out using standard techniques (Sive et al., 2000). Eggs from albino mothers were used for the TUNEL experiments.

### RNA Synthesis

Plasmid constructs encoding Geminin^WT^, Geminin mutants, Cdt1^WT^, Cdt1^NGB^, Cdt1^C479^, Cdc25, Cdc25^SA^, Cdc2^WT^, and Cdc2^AF^ were previously described [22], [28], [36], [37]. A plasmid encoding Chk1^DA^ was kindly provided by Noriyuki Sagata [62]. Plasmids encoding Chk2^WT^ and Chk2^DN^ were kindly provided by Jill Sible [48]. Chk2^DN^ has previously been shown to have dominant-negative effects when over-expressed in Xenopus embryos [48], [63]. For in vitro RNA synthesis, plasmid constructs were linearized with Not I and transcribed with SP6 or T7 polymerase in the presence of ribonucleotide triphosphates and diguanosine triphosphate cap (m^7^GpppG, GE Healthcare) according to protocols provided by the supplier of the enzyme (Promega). RNAs were resuspended and diluted in water before injection.

### Microinjection

Embryos were injected at the two-cell stage with a volume of 10 nl per cell. To deplete Geminin, embryos were injected with 16 ng per cell of an equal mixture of anti-Geminin H and anti-Geminin L morpholino oligos (Gene Tools LLC) as previously described [28]. For some experiments, 2.5 ng of anti-Geminin H oligo was injected per cell as described [35]. To express proteins, embryos were injected with 30–8000 pg of synthetic RNA per cell. The amounts injected were as follows: Geminin and Geminin mutants 30 pg/cell; Cdt1 and Cdt1 mutants 15–500 pg/cell; Cdc2^AF^ and Cdc25^S287A^ 150 pg/cell; Chk1^DA^ 5000 pg/cell; and Chk2^DN^ 5000–7500 pg/cell. Morpholino oligos and RNAs were injected separately.

### Real Time PCR

Five embryos from the same clutch of eggs that had been identically treated were pooled for each measurement. Embryos were homogenized in RNA extraction buffer (50 mM Tris-HCl pH 7.5, 50 mM NaCl, 5 mM EDTA, 0.5% SDS) and treated with 0.4 mg/ml protease K for 30 min at 45°C followed by phenol/chloroform extraction. Nucleic acids were ethanol precipitated and resuspended in Transcription Buffer (Promega) containing 0.8 mM fresh DTT and 6 U RNAsin (Promega). DNA was digested by adding 1 U RQ1 DNAse (Promega) and incubating at 37°C for 30 minutes. The samples were re-extracted with phenol/chloroform, re-precipitated with ethanol, and resuspended in water. cDNA synthesis was carried out using a standard kit (Ambion) and RT-PCR was performed using an Applied Biosystems 7500 Fast Real Time PCR System. Primers and fluorescently labeled probes for RT-PCR were designed using Primer Design software (Applied Biosystems). All RNA levels were normalized to the amount of 18S ribosomal RNA in each sample and expressed as a percentage of the RNA level in control embryos from the same mother. Animal caps were dissected at stage 8 [64] and cultured in MBSH (110 mM NaCl, 2 mM KCl, 1 mM MgSO_4_, 2 mM NaHCO_3_, 0.5 mM sodium phosphate, 14 mM Tris pH 7.6) containing 0.1% BSA, with or without 20 ng/ml Human Activin A (Sigma). RNA was isolated when companion embryos reached the early gastrula stage (Nieuwkoop stage 10.5).

### Statistical Analysis

In all cases where results were analyzed statistically, at least three independent experiments were performed. An independent experiment is one where the embryos came from a separate mother. The expression of each gene was calculated as a percentage of the expression level in control embryos from the same mother. P values were calculated using Student's paired t-test; paired samples came from the same mother.

### *In situ* hybridization

In situ hybridization with digoxigenin-labeled probes and X-gal (5-bromo-4-chloro-3-indolyl-β-D-galactopyranoside) staining were performed using standard techniques [65], except that treatment with RNases A and T1 was omitted. Plasmids encoding antisense probes for Xbra and Gsc were kindly supplied by Sergei Sokol [66], [67]. DIG-RNA labeling mix, alkaline phosphatase-conjugated anti-Digoxigenin Fab fragments, and BM-purple AP precipitating substrate were purchased from Roche. Stained embryos were scored blindly to avoid biasing the results.

### TUNEL Staining

TUNEL staining was performed using published techniques [68], [69], except that immediately after fixation and rehydration the embryos were rinsed in antigen retrieval buffer (10 mM sodium citrate pH 6.0), steam heated to 100°C for 30 minutes in a double boiler, and slowly cooled. As a positive control, embryos were treated with DNAse I (1 U/µl in 50 mM Tris-HCl pH 7.5, 1 mg/ml BSA) for 30 minutes at room temperature in order to induce double stranded breaks. As a negative control, the terminal deoxynucleotidyl transferase (TdT) enzyme (Invitrogen #10533-065) and the digoxigenin-dUTP (Roche #11 093 088 910) were left out of the reaction.

### Antibodies

Antibodies raised against Xenopus Geminin and Cdt1 were described previously [22], [37]. Phosphospecific antibodies against pS^139^ H2A.X and pS^345^ Chk1 were purchased from Cell Signaling. Antibodies against Xenopus Chk2 were kindly supplied by Akiko Kumagai and William Dunphy [45], and antibodies against phospho-T^383^ Chk2 were purchased from Abcam.

## Supporting Information

Figure S1 **Neither Geminin Depletion nor Bypass of the DNA Replication Checkpoint causes Increased Apoptosis.** Two-cell embryos were left uninjected (A, B), treated with hydroxyurea (E), or injected on one side with anti-Geminin MOs or RNA encoding with Cdt1^NGB^, Cdc2^AF^, Cdc25^SA^, Chk1^DA^, or Chk2^DN^ (C–D, F–I). RNA encoding β-galactosidase was co-injected as a lineage tracer. When the embryos reached stage 10.5 they were fixed and stained for β-galactosidase activity using X-gal (blue) and for apoptotic cells using the TUNEL reaction (purple). (A) Positive control (embryos pre-treated with DNase I); (B) Negative control (TdT and labeled nucleotide omitted from the reaction). Some embryos showed a faintly positive TUNEL reaction even on the uninjected side (F, I).
(TIF)

Figure S2 **Bypass of the DNA Replication Checkpoint causes a General Loss of Zygotic Transcription.** Both cells of a 2-cell embryo were injected with RNA encoding Cdc2^AF^ in order to bypass the DNA Replication Checkpoint. When the embryos reached stage 10.5, the expression of Xnr5, Gsc, Xbra, Xwnt8, and Zic3 was measured by RT PCR. Asterisk indicates P<0.05; double asterisk indicates P<0.01.
(TIF)

Figure S3 **Partial Depletion of Geminin causes a Phenotype Similar to that of Complete Depletion.** Two-cell embryos were injected on both sides with either 2.5 ng or 16 ng of anti-Geminin MOs/side. At stage 10.5, protein levels were determined by immunoblotting (left panel) and RNA levels were measured by RT PCR (right panel).
(TIF)
