# Supplementary figures and images for: Geminin Is Required for Zygotic Gene Expression at the Xenopus Mid-Blastula Transition
Source: PLoS One. 2012 May 25;7(5):e38009. doi: 10.1371/journal.pone.0038009 (PMC3360639; doi:10.1371/journal.pone.0038009)

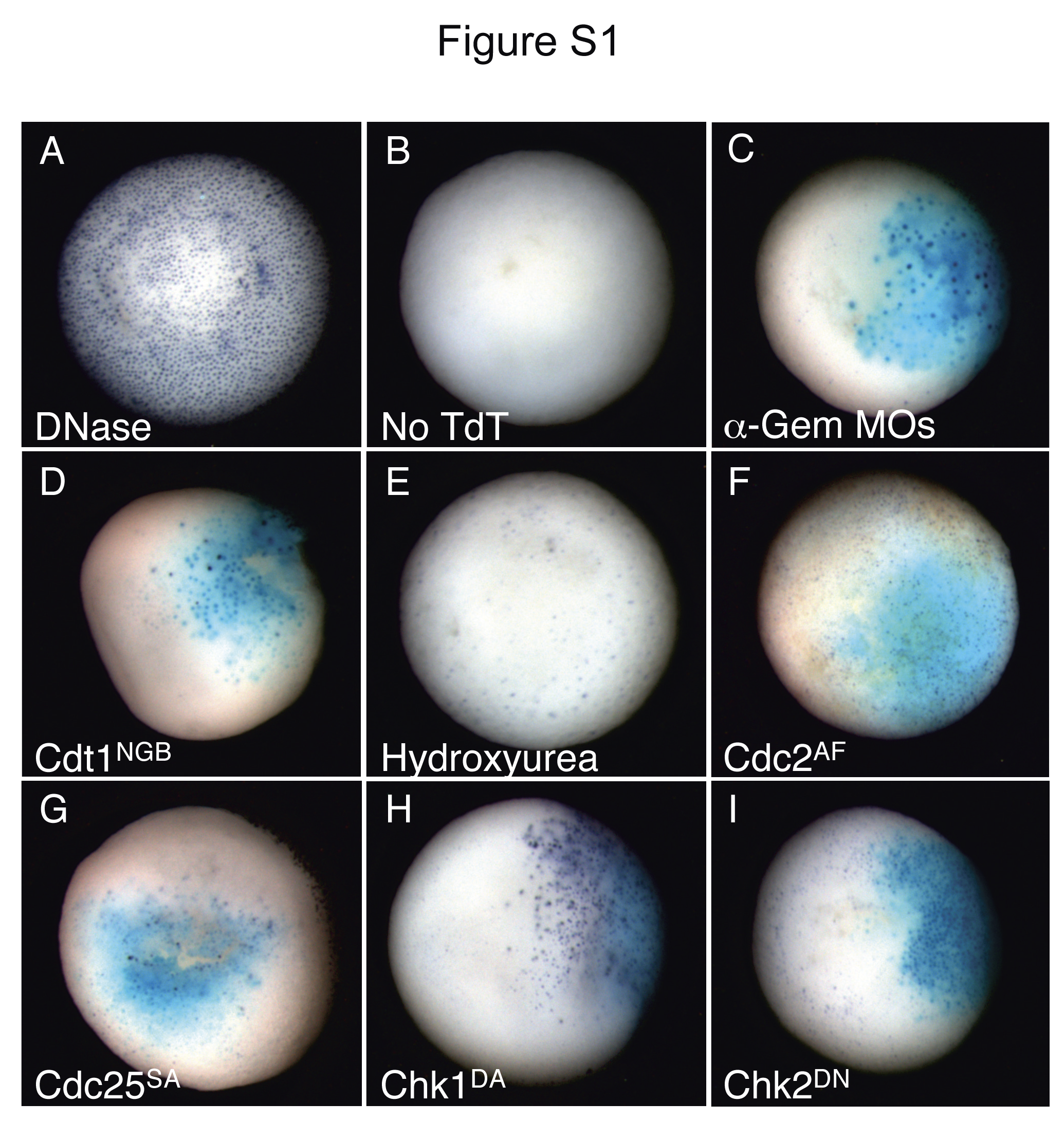

Supplement: Figure S1 — Neither Geminin Depletion nor Bypass of the DNA Replication Checkpoint causes Increased Apoptosis. Two-cell embryos were left uninjected (A, B), treated with hydroxyurea (E), or injected on one side with anti-Geminin MOs or RNA encoding with Cdt1NGB, Cdc2AF, Cdc25SA, Chk1DA, or Chk2DN (C–D, F–I). RNA encoding β-galactosidase was co-injected as a lineage tracer. When the embryos reached stage 10.5 they were fixed and stained for β-galactosidase activity using X-gal (blue) and for apoptotic cells using the TUNEL reaction (purple). (A) Positive control (embryos pre-treated with DNase I); (B) Negative control (TdT and labeled nucleotide omitted from the reaction). Some embryos showed a faintly positive TUNEL reaction even on the uninjected side (F, I). (TIF) [file pone.0038009.s001.tif]

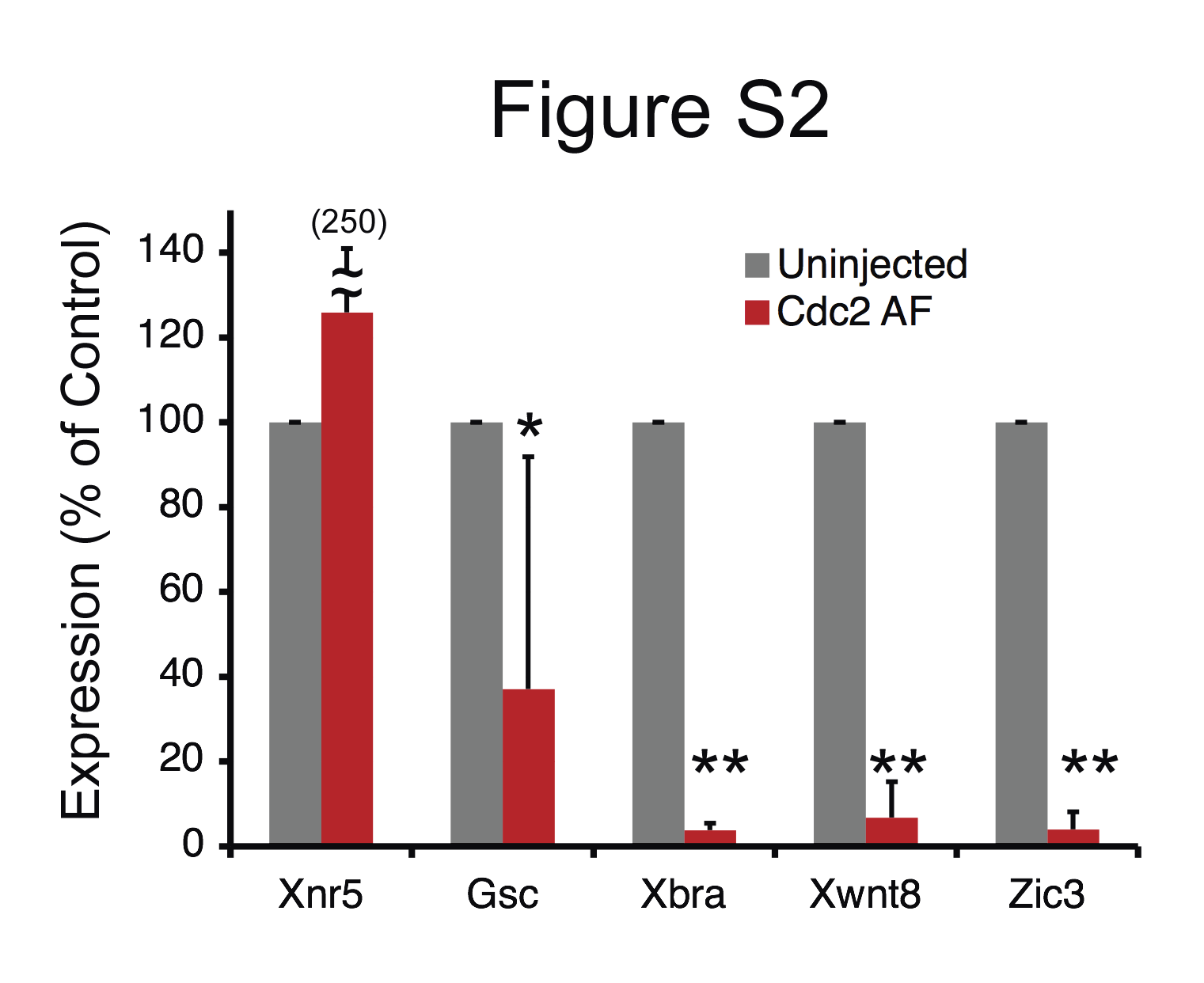

Supplement: Figure S2 — Bypass of the DNA Replication Checkpoint causes a General Loss of Zygotic Transcription. Both cells of a 2-cell embryo were injected with RNA encoding Cdc2AF in order to bypass the DNA Replication Checkpoint. When the embryos reached stage 10.5, the expression of Xnr5, Gsc, Xbra, Xwnt8, and Zic3 was measured by RT PCR. Asterisk indicates P<0.05; double asterisk indicates P<0.01. (TIF) [file pone.0038009.s002.tif]

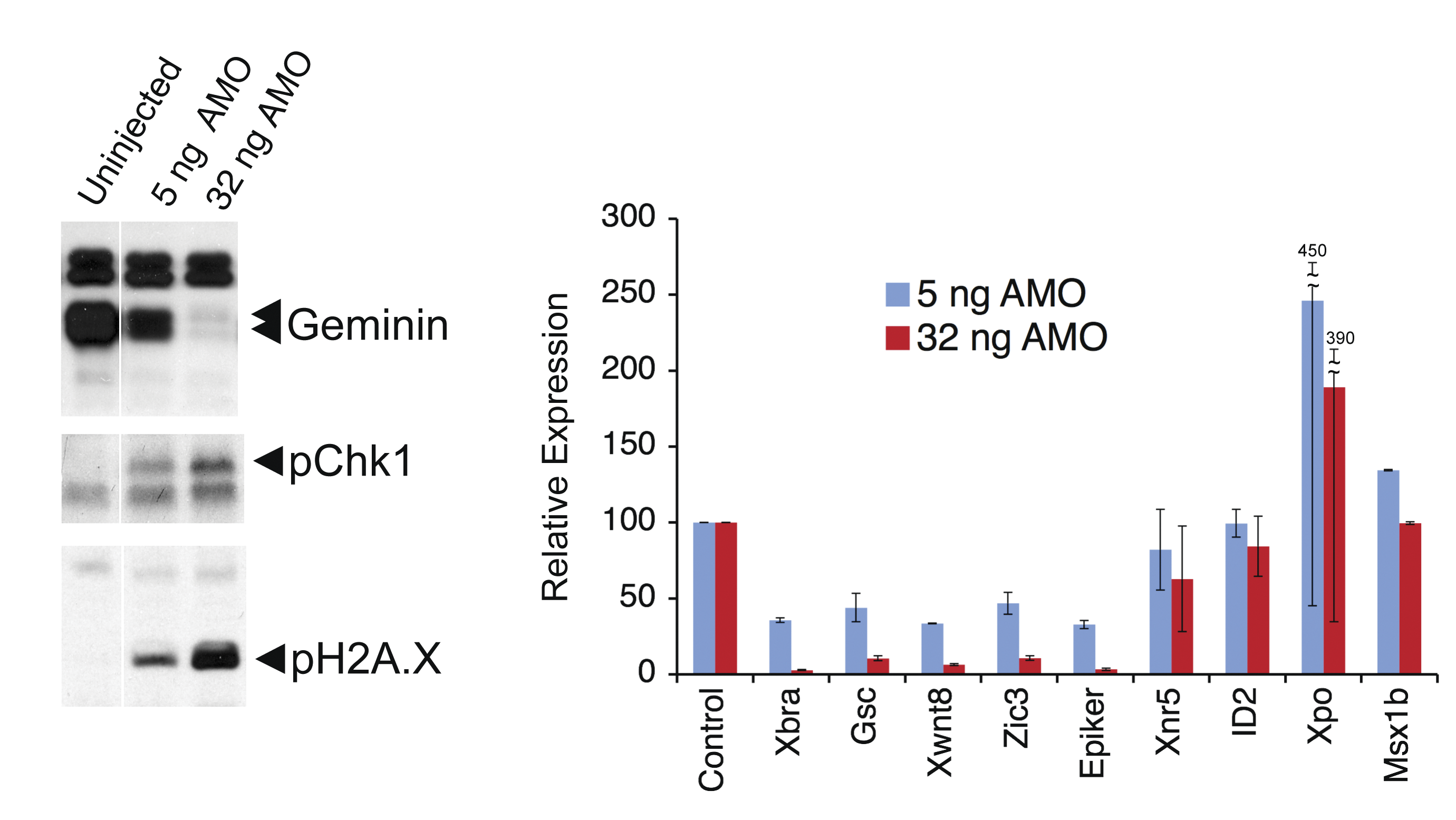

Supplement: Figure S3 — Partial Depletion of Geminin causes a Phenotype Similar to that of Complete Depletion. Two-cell embryos were injected on both sides with either 2.5 ng or 16 ng of anti-Geminin MOs/side. At stage 10.5, protein levels were determined by immunoblotting (left panel) and RNA levels were measured by RT PCR (right panel). (TIF) [file pone.0038009.s003.tif]
